# Causal relationship between novel antidiabetic drugs and ischemic stroke: a drug-targeted Mendelian randomization study

**DOI:** 10.3389/fcvm.2024.1449185

**Published:** 2024-09-24

**Authors:** Zongliang Yu, Xinyi Liu, Xue Feng, Xiaonan Zhang, Rui Gao

**Affiliations:** ^1^ Beijing University of Chinese Medicine, Beijing, China; ^2^ Xiyuan Hospital, China Academy of Chinese Medical Sciences, Beijing, China

**Keywords:** ischemic stroke, novel antidiabetic agents, GLP-1 receptor agonists, SGLT-2 inhibitors, DPP-4 inhibitors, Mendelian randomization analysis

## Abstract

**Background:**

The escalating global economic burden of ischemic stroke poses a significant public health challenge amid global aging trends. The broad therapeutic efficacy of new antidiabetic drugs may offer new options in the prevention and treatment of ischemic stroke. Consistent conclusions regarding the relationship between novel antidiabetic agents and the risk of ischemic stroke remain elusive, and the causal relationship deserves further investigation.

**Materials and methods:**

Three novel antidiabetic drug targets were selected, and cis-expression quantitative trait loci (cis-eQTL) were screened as instrumental variables. Genetic association data for ischemic stroke were obtained from the Genome-wide Association Study (GWAS) database. Mendelian randomization (MR) analysis, facilitated by R software, calculated MR estimates for each single nucleotide polymorphism (SNP), and meta-analysis was performed using five methods. To ensure robustness, sensitivity analyses, heterogeneity analyses, horizontal pleiotropy analyses, and co-localization analyses were conducted for significant MR associations.

**Results:**

Three eQTLs for antidiabetic drug genes served as instrumental variables, utilizing a GWAS dataset comprising 34,217 cases and 406,111 controls for ischemic stroke. Genetic variants in glucagon-like peptide-1 receptor agonists (GLP-1 RA) targets exhibited a positive correlation with ischemic stroke risk (OR 1.06, 95% CI 1.04–1.08, *P* = 0.000), while genetic variation in dipeptidyl peptidase 4 inhibitors (DPP-4i) targets showed a negative association with ischemic stroke risk (OR 0.93, 95% CI 0.89–0.97, *P* = 0.003). Sensitivity analyses supported robust conclusions, revealing no heterogeneity or horizontal pleiotropy.

**Conclusion:**

This study found that GLP-1 RA and DPP-4i were associated with an increased risk of ischemic stroke by MR analysis. Although sensitivity analyses provide support for this result, it contradicts previous knowledge. Therefore, the results of this study still need to treated with caution. Updated and more in-depth GWAS data and high-quality real-world data are expected to validate the results.

## 1 Introduction

Ischemic stroke, constituting approximately 80% of all stroke cases (1), exhibits an age-associated surge in incidence. Projections suggest a global increase to 89.32 per 100,000 people by 2030 (2). While strides have been made in mitigating ischemic stroke lethality, survivors contend with enduring disabilities impacting daily life (3). This scenario amplifies the escalating global economic burden, posing a substantial public health challenge amid global aging trends (4). The current gold standard for acute ischemic stroke treatment involves intravenous thrombolysis and thrombectomy. However, the efficacy is confined to a small subset of patients due to restricted treatment limitations (5). Concurrently, secondary brain tissue damage induced by ischemia-reperfusion persists as a significant concern (6). Therefore, primary prevention before the onset, encompassing strategies like antiplatelet agents, statins, and blood pressure control, emerges as the most effective means to curtail the harm and disease burden of ischemic stroke (7).

Disturbed glucose metabolism stands out as a crucial risk factor for ischemic stroke (8), emphasizing the significance of maintaining moderate blood glucose levels in primary prevention (9, 10). In recent years, emerging novel antidiabetic agents such as glucagon-like peptide-1 receptor agonists (GLP-1 RA), sodium-glucose co-transporter 2 inhibitors (SGLT-2i), and dipeptidyl peptidase 4 inhibitors (DPP-4i) have demonstrated robust therapeutic effects across endocrine, cardiovascular, and renal domains (11–13). Their diverse therapeutic effects present potential options in ischemic stroke prevention and treatment. However, conclusive conclusions about the relationship between novel antidiabetic drugs and ischemic stroke risk remain elusive, warranting in-depth exploration of their effects and causality (14–16).

Hence, this study employs Mendelian randomization (MR) analysis to delve into the causal relationship between the use of novel antidiabetic drugs and the risk of ischemic stroke. Leveraging genetic variants associated with drug targets as instrumental variables, MR simulates a randomized controlled trial setting, offering insights into the reuse potential and risk of novel antidiabetic drugs in ischemic stroke. The outcomes hold promise as a reference for clinical practice.

## 2 Materials and methods

### 2.1 Identification of target data for novel antidiabetic drugs

Novel antidiabetic drugs, including GLP-1 RA, SGLT-2i, and DPP-4i, were selected for this study. Genes encoding the target proteins of these drugs were meticulously identified from the Drugbank (v5.0) and ChEMBL (v29.0) databases (17, 18). The chromosomal locations, start, and termination sites of these genes were precisely determined through the National Center for Biotechnology Information (NCBI).

### 2.2 Identification of cis-expression quantitative trait loci (eQTL) data associated with novel antidiabetic drug targets

Given the proximity of cis-eQTL to target genes in drug development studies (19, 20), we extracted cis-eQTL with full statistical significance (false discovery rate <0.05 ± 1Mb per probe) from the eQTLGen consortium database (21). This approach aims to analyze the genetic underpinnings of complex traits through blood gene expression.

### 2.3 Genetic association data screening for ischemic stroke

Genetic association data for ischemic stroke were obtained from the European Ischemic Stroke Cohort, the largest GWAS meta-analysis of the disease to date (22), pooled by the MEGASTROKE Consortium. The pooled data were adjusted for unknown confounders such as sex and ethnicity to avoid possible bias. The type of stroke included any type of ischemic stroke, and a total of 34,217 cases and 406,111 controls were included (23). All ischemic stroke diagnoses were confirmed by clinical symptoms and imaging criteria. All participants gave informed consent, and the local research ethics committee and institutional review board approved the study.

### 2.4 MR analysis

The TwoSampleMR R software package (v 4.3.2) was used for MR analysis. To ensure the reliability of the results, stringent criteria were used to filter low-quality genetic tools. Based on the key assumption that MR analyses are established (24), the criteria for screening SNPs in the exposure data were as follows (25): (1) genome-wide SNPs with significance (*P* < 5 × 10^−6^), (2) exclusion of weak instrumental variables with an F-statistic <10, (3) linkage disequilibrium (LD) testing to ensure independence of selected instrumental variables (r^2 ^< 0.1 within a 1,000 kb range) (26, 27), and (4) removal of SNPs with incompatible or palindromic allele frequencies. Subsequently, drug target-associated SNPs were further screened based on gene chromosomal loci ±100 kb range and filtered based on eaf >0.01. Finally, the ending data were extracted and merged based on the filtered instrumental variables.

MR estimates for each SNP were calculated using the Wald ratio method, and meta-analysis of MR estimates was performed using inverse variance weighted (IVW), MR-Egger, weighted median, simple mode, and weighted mode (28). IVW assumes that each genetic variant exists independently and can only influence the outcome through the exposure of interest (29). However, in the presence of pleiotropy, causality may be biased. Methods such as MR Egger and weighted median may avoid the bias caused by pleiotropic effects and confounders in genetic variants, but may yield wider confidence intervals (30, 31). The IVW method has been reported to be slightly more accurate than the other methods under practical conditions (28). Therefore, the results of this study are mainly based on the IVW method, with the other four methods as its complement. MR estimates with *P* < 0.05 were considered statistically significant. All estimates were expressed as odds ratio (OR) with a 95% confidence interval (CI) per standard deviation increase in the corresponding exposure.

### 2.5 Sensitivity and co-localization analysis

The MR-PRESSO test was used to test for horizontal pleiotropy. If horizontal pleiotropy was found between instrumental variables, outliers were removed and the MR analysis was re-executed (32). Sensitivity analyses were conducted via the leave-one-out method. This method calculates the meta effect of the remaining SNPs by progressively eliminating each SNP in order to observe the potential impact of each SNP on the study results (33). Cochran's Q statistic was utilized to assess potential heterogeneity. The method infers the presence of heterogeneity in the sample by calculating the estimated difference between the causal effect estimate and the estimated difference in the instrumental variable (34). The presence of horizontal pleiotropy can cause the results of MR analyses to be untenable, so we performed the MR Egger intercept test in addition to the MR-PRESSO test (30). The above analysis was done using TwoSampleMR R package (v 4.3.2).

Subsequently, co-localization analysis was executed with the colocR package and default prior. For the eQTL dataset, *a priori* probabilities of 1E-04 for cis-eQTL and ischemic stroke associations were set, with the prior probability of a single variant affecting both traits at 1E-05. Significant co-localization was determined at PH4 >0.80, and genes strongly co-localized with ischemic stroke were considered as potential target molecules. The overall design of the study is shown in Supplementary Figure 1.

## 3 Results

### 3.1 Selection of genetic tools for novel antidiabetic drugs

We identified three major genes encoding proteins experimentally modified by novel antidiabetic drugs. To generate genetic tools for substituting novel antidiabetic drug targets, we selected cis-eQTL within ±100 kb of each gene's genomic location (Table 1). The eQTL of the three antidiabetic drug genes with the highest significance were chosen as tool variables based on their *P* values. GWAS data for ischemic stroke were obtained from the ebi-a-GCST005843 dataset (22), comprising 34,217 cases and 406,111 controls from the MEGASTROKE Consortium, encompassing a total of 7,537,579 SNPs.

**Table 1:** Target genes and cis-eQTL of antidiabetic drugs.

Drug class	Encoding genes of target proteins		Gene location
	DrugBank	ChEMBL	
GLP-1 RA	GLP1R	GLP1R	Chr6: 39,016,557–39,059,079
SGLT-2i	SLC5A2	SLC5A2	Chr: 16: 31,494,444–31,502,090
DPP-4i	DPP4	DPP4	Chr2: 162,848,755–162,930,725

### 3.2 MR analysis

**Figure 1:**
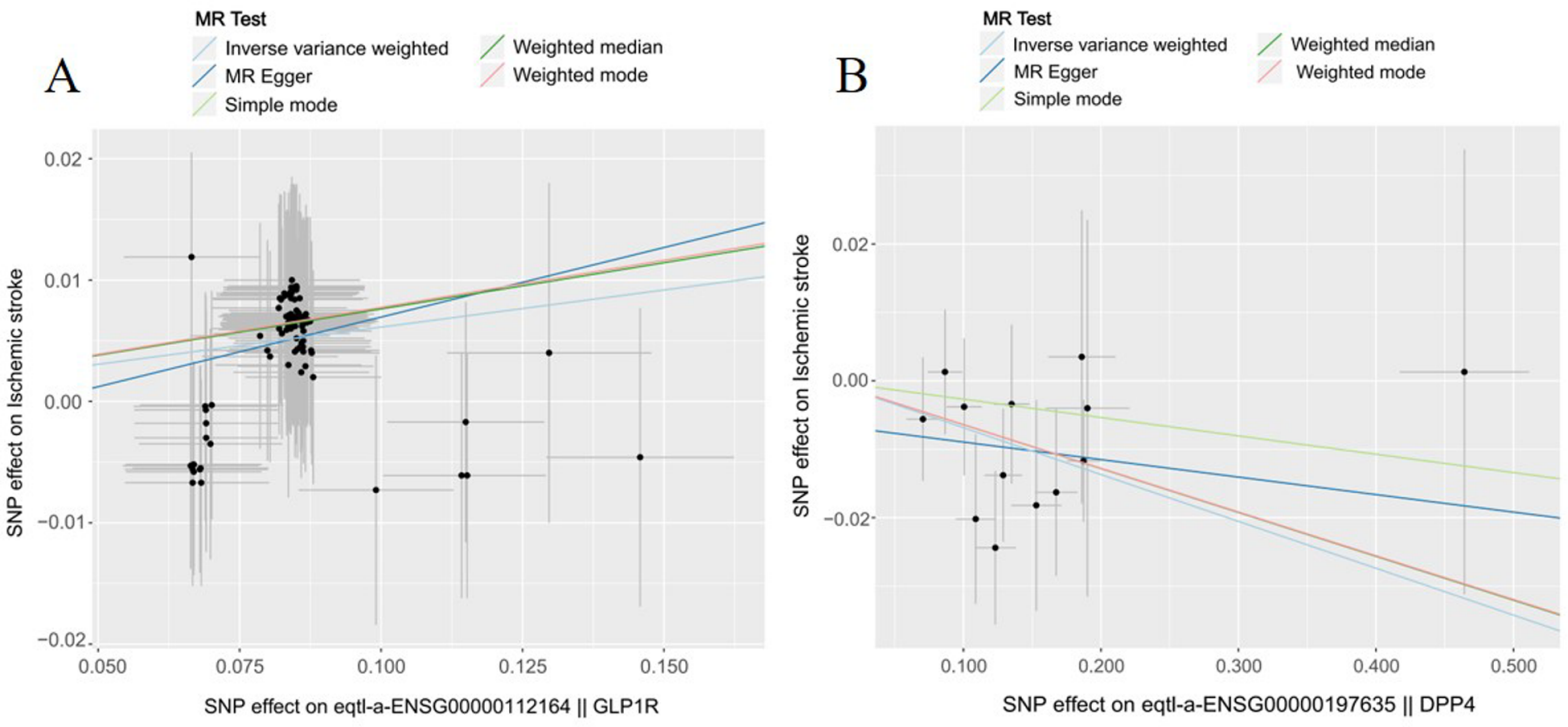
The effect of genetic variation in novel antidiabetic drug targets on ischemic stroke. **(A)** GLP1R **(B)** DPP4.

As shown in Table 2, GLP1R were significantly associated with ischemic stroke risk according to the Inverse variance weighted method (OR 1.06, 95%CI 1.04–1.08, *P* = 0.000). Additionally, DPP4 were significantly associated with ischemic stroke risk (OR 0.93, 95%CI 0.89–0.97, *P* = 0.003). Conversely, SLC5A2 was not statistically significantly associated with ischemic stroke risk (OR 1.23, 95%CI 0.97–1.55, *P* = 0.077). The scatter plot (Figure 1) visualized the causal relationship. Each point in the graph represents a SNP, and the short lines of the cross at each point reflect its 95% CI. The abscissa is the effect of the SNP on the exposure, and the ordinate is the effect of the SNP on the outcome. The slash lines of different colors represent the MR fitting results of different calculation methods. A slope greater than 0 indicates that the exposure factor is a disadvantage of ischemic stroke. For the fitting results of different methods, the results of IVW are generally the main ones. All five pooling methods concurred on the ischemic stroke risk of GLP1R and the protective effect of DPP4.

**Table 2:** Estimated effects of genetic variations in antidiabetic drug targets on ischemic stroke.

Gene	Method	*N* SNPs	β	OR	*P* value
GLP1R	MR Egger	113	0.115	1.120	0.204
	Weighted median	113	0.077	1.079	0.000*
	IVW	113	0.061	1.063	0.000*
	Simple mode	113	0.078	1.081	0.014*
	Weighted mode	113	0.078	1.081	0.015*
SLC5A2	Wald ratio	1	0.208	1.232	0.077
DPP4	MR Egger	13	−0.026	0.975	0.694
	Weighted median	13	−0.064	0.938	0.046*
	IVW	13	−0.068	0.934	0.003*
	Simple mode	13	−0.027	0.974	0.605
	Weighted mode	13	−0.064	0.938	0.135

N SNPs represents the number of SNPs; OR, odds ratio; IVW, inverse variance weighted, MR, mendelian randomization.

* *P* value meets the significance threshold.

### 3.3 Sensitivity analysis and co-localization analysis

We performed the sensitivity analysis by the “leave-one-out” method (Supplementary Figure 2). The results showed that excluding each SNP individually did not affect the results of the MR pooling analysis, which indicated that the stability of the MR analysis.

To further verify the robustness of MR analysis, we analyzed the heterogeneity of MR by Cochran's Q statistic, and the MR-PRESSO method and Egger intercept test verified the horizontal pleiotropy of MR. As shown in Table 3, the results all indicated that there was no heterogeneity or horizontal pleiotropy between GLP1R and DPP4 gene and ischemic stroke risk (*P* > 0.05).

**Table 3:** Heterogeneity analysis and pleiotropy analysis.

Outcome	Gene	*P* for MR Egger test	*P* for Q test	*P* for MR-PRESSO test
Ischemic stroke	GLP1R	0.551	1.000	1.000
	DPP4	0.482	0.877	0.904

**Figure 2:**
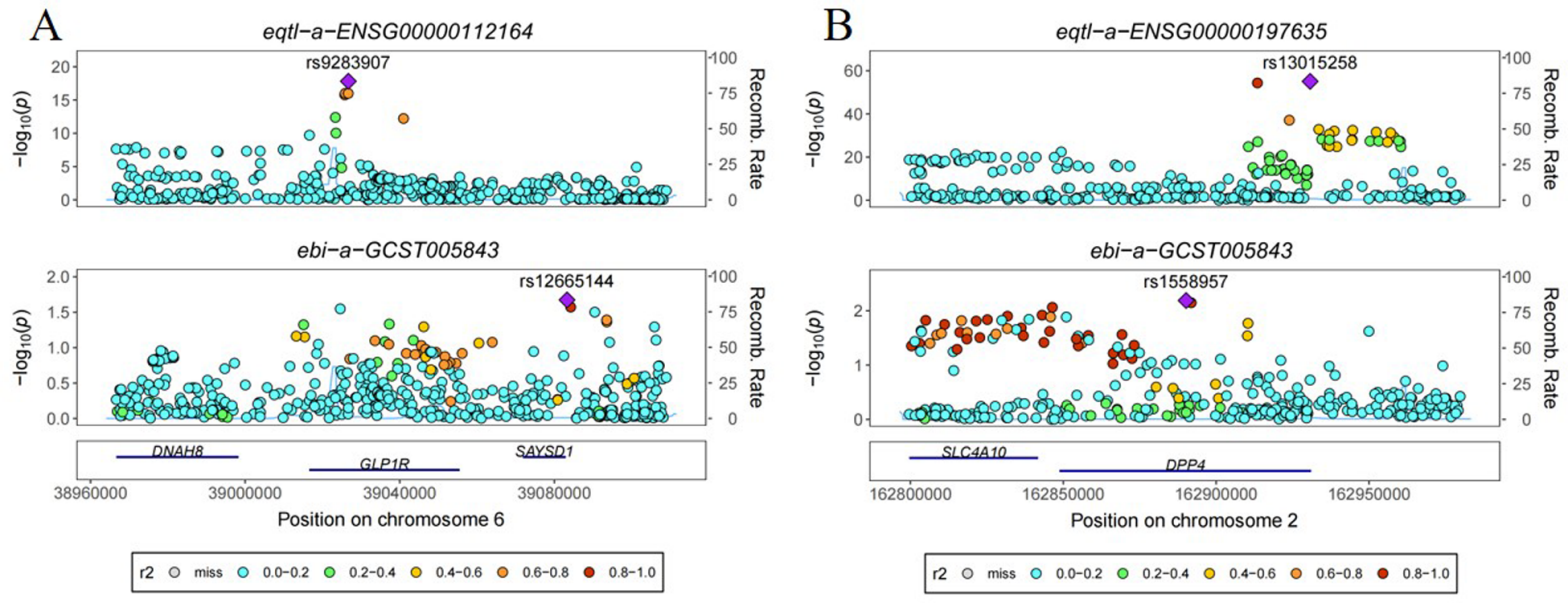
Co-localization analysis of the effect of genetic variants in novel antidiabetic drug targets on ischemic stroke. **(A)** GLP1R **(B)** DPP4.

Co-localization analyses were conducted to ascertain the probability of shared causal genetic variants between SNPs associated with ischemic stroke and novel antidiabetic drug eQTL. Results revealed a 6.5% probability of the GLP1R gene having a shared genetic effect between eQTL and ischemic stroke risk, while the DPP4 gene exhibited a 10.7% probability of shared genetic effect between eQTL and ischemic stroke risk (Figure 2).

## 4 Discussion

In recent years, novel antidiabetic agents, including GLP-1 RA, SGLT-2i, and DPP-4i, have exhibited broad therapeutic efficacy, potentially introducing new avenues for ischemic stroke prevention and treatment (35). However, the relationship between novel antidiabetic drugs and ischemic stroke risk lacks consistent conclusions. This study marks the inaugural exploration of the causal association between genetic variants of novel antidiabetic drug targets and ischemic stroke risk through MR analysis. Leveraging large-scale genetic association data on ischemic stroke, our analyses unveil a positive association between genetic variants of GLP-1 RA targets and ischemic stroke risk, contrasting with a negative association for genetic variants of DPP-4i targets. Notably, since GLP-1 RA promotes gene expression, and DPP-4i is an inhibitor of gene expression. Therefore, the final result of the analysis is that both GLP-1 RA and DPP-4i are risk factors for ischemic stroke. Although this result is somewhat contradictory to common sense, rigorous sensitivity analyses, heterogeneity assessments, and horizontal pleiotropy analyses support the robustness of these findings.

GLP-1 RA effectively lowers blood glucose in type 2 diabetes patients by stimulating β- and α-cell GLP-1 receptors, enhancing insulin secretion, inhibiting glucagon secretion, and improving insulin sensitivity (36). A meta-analysis of seven large randomized controlled studies with cardiovascular outcomes as endpoints showed that GLP-1 RA was able to reduce the risk of total stroke by 16% (37). This seems to provide compelling clinical evidence for the protective effect of GLP-1 RA against ischemic stroke. However, in all published studies, the subjects were clearly type 2 diabetic and the incidence of stroke was only one of the secondary indicators. For other patients with other metabolic diseases who may be taking GLP-1 RA for various reasons or for the normal population, there are no clear conclusions about the role of the drug in ischemic stroke. Furthermore, even in studies of patients with type 2 diabetes, the role of GLP-1 RA on stroke remains contradictory (38). For example, in the ELIXA study, it was found that GLP-1 RA appeared to increase the incidence of multiple strokes (39). Similarly, some early findings seem to suggest a protective effect of DPP4i against stroke (40, 41). However its possible risks are yet to be followed up and discussed in real-world study results.

Another conflicting finding came from a previously published Mendelian randomization study (42). This study found that genetic variants in GLP-1 were not associated with ischemic stroke, which seems to be inconsistent with both the previous study and our findings. The reason for this contradiction is mainly related to the limitation of GWAS sequencing depth and the number of SNPs (43). When the number of available SNPs is less than three, limiting the scope of a full Mendelian randomization (MR) analysis, these results should be excluded from the analysis to ensure the robustness and reliability of the results (44, 45). Therefore, the conclusions of the present study can be considered as a complement and extension of the results of that study.

Overall, our study identified GLP-1 RA and DPP-4i as risk factors for ischemic stroke.The MR analysis method was able to avoid confounding factors such as blood glucose levels and population, thus obtaining an exact causal association in a larger population. In addition, co-localization analysis of MR results was performed for the first time in this study, although this result does not seem to be very favorable. This apparent inconsistency may stem from the methodological differences between the two approaches. MR selectively identifies exposure-related variants, whereas co-localization is more conservative, requiring significant associations of causal variants with both traits. The co-localization analysis was performed with a Bayesian test, which assumes that there is only one shared causal variance locus. If more than one shared causal variance locus exists within an association interval, the results of the analysis may be affected (46, 47). In addition, some statistical experts believe that a higher PH4 suggests the presence of horizontal pleiotropy between the two, and therefore a lower PH4 can instead more favorably demonstrate a causal relationship between the two phenotypes (48).

SGLT-2i operates by selectively inhibiting glucose reabsorption in proximal renal tubules, leading to increased urinary glucose excretion (49, 50). Studies have demonstrated its efficacy in reducing the risk of hospitalization for ischemic stroke and improving cardiovascular outcomes, including ischemic stroke (16, 51). A retrospective cohort study indicated a lower risk of new stroke in SGLT-2i users compared to non-users, with an even greater reduction in patients concurrently using other hypoglycemic and lipid-lowering medications (52). Experimental studies further supported the neuroprotective effects of cagliflozin in a mouse model of moderate ischemic injury, reducing infarct volume, brain swelling, and improving neurological function (53). However, conflicting evidence suggests that SGLT-2i may not significantly reduce the incidence of ischemic stroke (14, 15). A meta-analysis involving five large randomized controlled trials with 46,969 subjects revealed no significant efficacy of SGLT-2i against fatal stroke, nonfatal stroke, ischemic stroke, or transient ischemic attack, with potential protective effects only against hemorrhagic stroke (54). Consequently, no consistent conclusions have been drawn regarding the relationship between SGLT-2i and the risk of ischemic stroke. This study, employing drug target-related genetic variants as instrumental variables, aims to infer causality by simulating a randomized controlled trial setting, offering a fresh perspective on addressing this issue. Our findings, however, fail to establish a causal association between gene variants in the target of action of SGLT-2i and the risk of ischemic stroke. It is noteworthy that due to the limitation of sequencing depth, we obtained an insufficient number of SNPs in the SGLT gene, and the results need to be interpreted with caution.

To the best of our knowledge, this represents the inaugural MR analysis on the target of action of novel antidiabetic drugs and the risk of ischemic stroke, providing a novel lens for drug development in this context. The strength of the MR design is its ability to infer causality while reducing confounding bias and reverse causality. Focusing on European ancestry minimizes spurious associations due to population stratification, and the selection of genetic variants as instruments within a narrow window of encoding genes mitigates biases arising from off-target effects and downstream proteins as instrumental variables.

However, there are some limitations to this study. Firstly, the current analysis was limited to European ancestry, which limits the generalizability of the results to other races. In addition, while drug target screening methods are effective in localizing effectiveness at the gene level, this somewhat limits our capacity to screen for SNPs. Although the screening criteria for SNPs in this study were sufficiently rigorous to ensure the reliability of the findings, the results need to be interpreted with caution. Furthermore, although we screened the cis-eQTL with the most significant relationship with gene expression of drug targets as a tool. In some cases, changes in eQTL may not fully reflect changes in blood glucose levels. However, due to the limitation of GWAS sequencing depth, we were unable to employ downstream indicators of blood glucose such as fasting blood glucose level and glycated hemoglobin as genetic tools for detailed discussion and analysis. This to some extent limits the accuracy of our drug target screening using MR analysis, so we look forward to more updated GWAS studies to provide more in-depth analysis and discussion of the findings.

## 5 Conclusion

This study found that GLP-1 RA and DPP-4i were associated with an increased risk of ischemic stroke by MR analysis. Although sensitivity analyses provide support for this result, it contradicts previous knowledge. Therefore, the results of this study still need to treated with caution. Updated and more in-depth GWAS data and high-quality real-world data are expected to validate the results.

## Data Availability

The original contributions presented in the study are included in the article/Supplementary Material, further inquiries can be directed to the corresponding author.
