# Supplementary figures and images for: Causal relationship between novel antidiabetic drugs and ischemic stroke: a drug-targeted Mendelian randomization study
Source: Front Cardiovasc Med. 2024 Sep 24;11:1449185. doi: 10.3389/fcvm.2024.1449185 (PMC11458414; doi:10.3389/fcvm.2024.1449185)

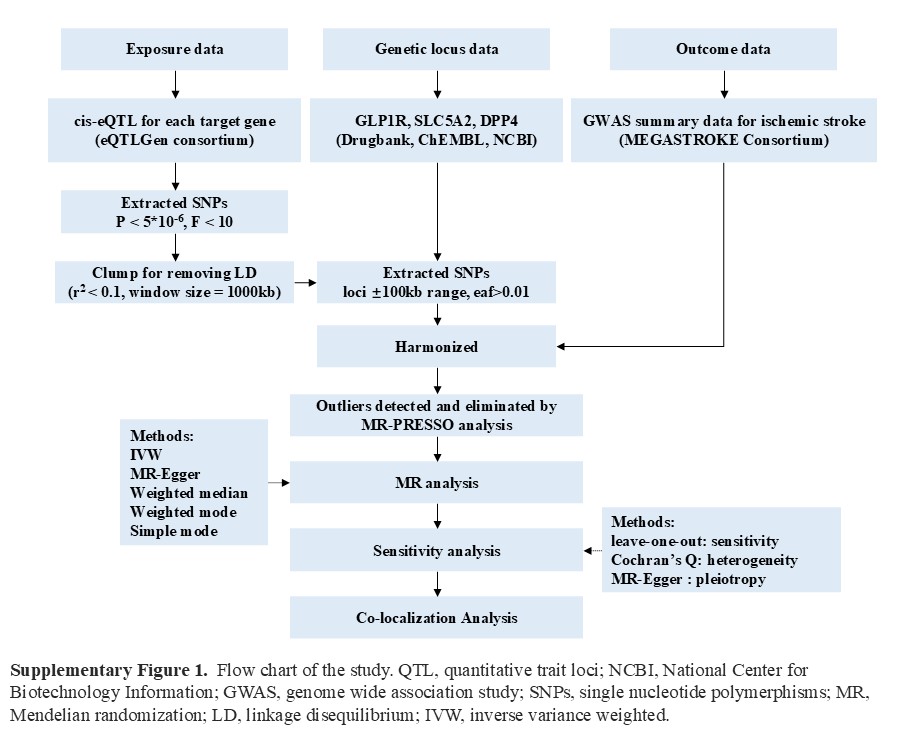

Supplement: Supplementary file 1 [file Image1.jpeg]

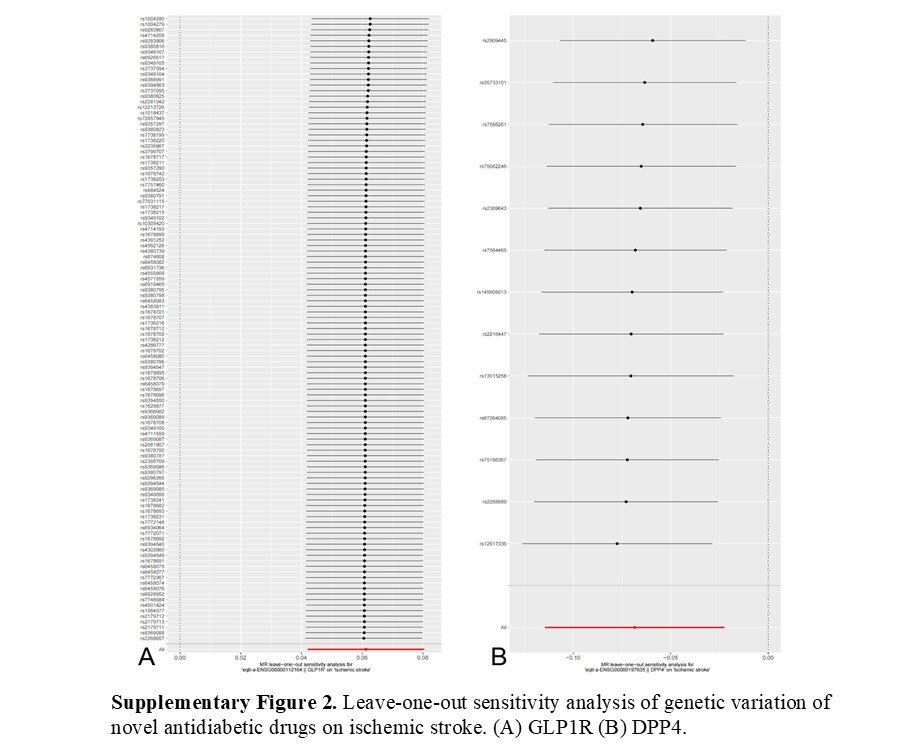

Supplement: Supplementary file 2 [file Image2.jpeg]
